# Scientific Concerns About Recent Fluid-Structure Interaction Models of the Aqueous Humor Outflow Pathway

**DOI:** 10.1167/iovs.66.15.18

**Published:** 2025-12-04

**Authors:** Mark Johnson, Darryl R. Overby, W. Daniel Stamer, C. Ross Ethier

**Affiliations:** 1 Biomedical Engineering Department, Northwestern University, Evanston, Illinois, United States; 2 Department of Bioengineering, Imperial College London, London, United Kingdom; 3 Department of Ophthalmology, Duke University, Durham, North Carolina, United States; 4 Department of Biomedical Engineering, Georgia Tech/Emory and Department of Ophthalmology, Emory University, Atlanta, Georgia, United States

**Keywords:** fluid mechanics, glaucoma, computational modeling, trabecular meshwork (TM), aqueous humor outflow

## Abstract

The development of sophisticated computational tools, combined with advanced ultrastructural imaging techniques, offers unprecedented opportunities to investigate aqueous humor outflow through the conventional pathway, the site of pathology responsible for ocular hypertension in glaucoma. Recently, a series of studies^1^^–^^7^ have used computational methods to study conventional outflow function. Regrettably, these studies^1^^–^^7^ appear to contain fundamental errors that lead to predictions that are inconsistent with established outflow physiology and raise concerns regarding methodology and apparent misrepresentations of published work. As a result, this body of work draws potentially misleading and erroneous conclusions about aqueous humor dynamics. It is therefore important to recognize and discuss these works to correct the archival record and avoid misdirecting future research.

## Physiological Inconsistencies

### The Velocity of Aqueous Humor Outflow Through the Trabecular Meshwork

The proximal part of the conventional outflow pathway includes the trabecular meshwork (TM) and Schlemm's canal. The velocity of aqueous humor outflow through the TM, and its most distal aspect, the juxtacanalicular connective tissue (JCT), is governed by three fundamental constraints: the aqueous humor turnover rate, anatomic dimensions of the conventional outflow pathway, and the physical law of mass conservation. Based on these constraints, the characteristic flow velocity through the human TM can be estimated to be approximately 10 µm/second.^a^ Whereas local variations may occur due to factors such as outflow segmentation or anatomic uncertainties, significant deviation from this estimate would be physiologically implausible, as mass conservation requires that flow velocities must circumferentially average to this value.

In one study from this series of papers, an average aqueous humor velocity of roughly 200 µm/second is shown in the JCT (fig. 5 of Ref. 5). A second study from the same group reports more physiological plausible velocities on the scale of 25 µm/second (fig. 7 from Ref. 7). Other papers from this group report lower velocities through this region (see the Table) but the deviations in flow velocity, spanning nearly two orders of magnitude (e.g., mid-scale values of 3 µm/seconds per figure 17 of Ref. 3 vs. 200 µm/second per fig. 5 of Ref. 5), were not mentioned or otherwise addressed in any of these publications.^2^^–^^5^^,^^7^

**Table.** Summary of Parameter Values Reported in References 1 to 5 and 7 (Columns 2–7) and Estimated Physiological Values (Column 8)

Parameter^*^	Karimi et al., 2022^7^	Karimi et al., 2022^2^	Karimi et al., 2023^1^	Karimi et al., 2023^5^	Karimi et al., 2024^4^	Karimi et al., 2024^3^	Estimated Physiological Values^†^
Typical TM/JCT velocity, µm/second^‡^	25^§^	10^‖^	N/A	**200** ^¶^	12^#^	3^**^	10
Pore velocity, µm/second	N/A	N/A	N/A	**130,000**	**110,000**	N/A	4000
Average wall shear stress in TM, Pa	**15** ^††^	**5** ^‡‡^	**0.8–2.3** ^§§^ **0.2–0.6** ^‖‖^	**3.2**	**3.1**	N/A	0.01
Reynolds number	N/A	N/A	N/A	**70**	**0.28**	**0.28**	≤0.002
Turbulence	**Turbulent**	Laminar	N/A	Laminar	Laminar	Laminar	Laminar

Reference 6 is not included in this table as these parameter values were not quantitatively reported in that paper.

Bolded values are not consistent with estimated physiological values.

N/A, not available.

* As of October 21, 2025, there were significant differences in the published values of the outflow velocities between the final journal-formatted versions of record for several references^2^^–^^4^^,^^7^ compared with the versions of those same manuscripts that appear on PubMed Central (PMC). Specifically, PMC versions reported velocities that were 100-fold higher,^7^ 1000-fold higher,^2^ or 10,000-fold higher^3^^,^^4^ than the velocities reported in the journal-formatted versions (cf. fig. 3 of Reference 2, fig. 17 of Reference 3, fig. 7 of Reference 4, or fig. 7 of Reference 7 between PMC and journal-formatted versions). We here use the lower reported values of the flow velocities as they appear in the journal-formatted versions.

† See text for details of how values in column 8 were determined, as based on physiological values.

‡ “Typical TM/JCT velocity” refers to the mid-point on the range of the reported velocity scale, except where otherwise stated.

§ From figure 7 of Reference 7.

‖ From figure 3 of Reference 2; there are typographic errors in two locations of Reference 2 where mm/second should be µm/second.

¶ From figure 5 of Reference 5.

# From figure 7 of Reference 4.

** From figure 17 of Reference 3.

†† From figure 8 from Reference 7.

‡‡ From figure 6 of Reference 2.

§§ Healthy eyes.

‖‖ Glaucomatous eyes.

To illustrate the consequence of supraphysiological outflow velocities, consider a TM flow velocity of 200 µm/second. Such a velocity would imply a total outflow rate of 50 µL/min^b^, a value that is 20-fold higher than the known aqueous production rate of 2.5 µL/minute. Whereas segmental outflow^10^ might account for some variability, reconciling such large differences would require only 5% of the TM to be actively involved in outflow filtration, a scenario incompatible with current understanding of outflow physiology.

It is notable that other reported quantities, such as the velocity of aqueous humor through the inner wall pores, shear stress, and Reynolds number, all of which depend directly on the outflow velocity, are also supraphysiological. We address these additional discrepancies below.

### The Velocity of Aqueous Humor Outflow Through Pores of the Inner Wall of Schlemm's Canal

The lowest porosities, and thus highest fluid velocities, within the conventional outflow pathway are located at the inner wall of Schlemm’s canal, where flow passes through micron-sized pores to cross an otherwise impermeable inner wall endothelium containing tight junctions.^11^^,^^12^ Using reported pore densities of 835 pores/mm^2^ and a pore diameter of 1.2 µm,^13^^,^^14^ the through-pore velocity is approximately 4000 µm/second^c^. This is in reasonable agreement with the estimate of 5000 µm/second provided by Braakman et al.^16^

In contrast, References 4 and 5 use a computational model to report an average through-pore fluid velocity of ∼110,000 µm/second or ∼130,000 µm/second, respectively. These values are approximately 30-fold larger than the above estimate of 4000 µm/second. The authors of References 4 and 5 support the velocities determined by their computational model by stating that other papers^13^^,^^14^^,^^16^^,^^17^ have reported velocities in inner wall pores of 100,000 to 950,000 µm/second. To be clear, those papers made no such claims. Instead, although it is difficult to be certain due to ambiguity in the writing, it appears^5^ that the through-pore velocities may have been miscalculated based on an erroneous interpretation of pore density, confusing pores/mm^2^ reported in References 13, 14, 16, and 17 with the *total* number of pores in an average human Schlemm’s canal. In other words, the through-pore velocities cited above and used to justify the computational results assume fewer than 300 pores along the entire inner wall of Schlemm's canal. This stands in contrast to the 835 pores per square millimeter of inner wall area reported by the references cited above.^13^^,^^14^

### Physiological Implications of Incorrect Aqueous Humor Velocities

Why does this matter? Here, we consider only the influence on two such predictions: hydrodynamic shear stress and “turbulence” within the outflow pathway.

#### Hydrodynamic Shear Stress

Regulation of intraocular pressure (IOP) requires that the conventional outflow pathway senses and responds to IOP-derived mechanical cues, such as hydrodynamic shear stress acting on cells within the TM and Schlemm’s canal.^18^^,^^19^ The shear stress is directly proportional to flow velocity (i.e. a 10-fold error in velocity will cause a 10-fold error in shear stress), and thus accurate estimates of aqueous humor velocity are critical to understand outflow physiology and IOP homeostasis.

Two of the aforementioned studies^4^^,^^5^ reported an average hydrodynamic wall shear stress of approximately 3.1 to 3.2 Pa in the intertrabecular spaces within the TM, whereas in the juxtacanalicular connective tissue,^1^ the reported wall shear stress were as high as “∼10.7 to 11.5 Pa and ∼12.2 to 14.5 Pa in the healthy and glaucoma cellularized models, respectively,” depending on which model was used. Likewise, figures showing the spatial distribution of hydrodynamic shear stress in the TM were plotted on scales up to 10 Pa (fig. 8 of Ref. 4 and fig. 6 of Ref. 5) or 30 Pa (fig. 12 of Ref. 1 and fig. 8 of Ref. 7). For context, these shear stress predictions exceed, by several-fold, the magnitude of the peak hydrodynamic shear stresses within the suprarenal aorta, typically 0.1 to 1 Pa.^20^ Thus, taken at face value, such simulations^1^^,^^4^^,^^5^^,^^7^ give the impression that TM cells reside in a hydrodynamic environment that is as demanding as that experienced by endothelial cells in large arteries. However, other models based on outflow velocities that are more aligned with current understanding of outflow physiology predict a hydrodynamic shear stress in the juxtacanalicular portion of the TM of approximately 0.01 Pa^d^, which is roughly 500-fold lower than the estimates from the aforementioned papers.^1^^,^^4^^,^^5^^,^^7^

#### Turbulence and the Reynolds Number

Turbulence is a characteristic feature of fast moving, inertially dominated flow that results in chaotic mixing and time-varying shear stress. Based on computational simulations, one study concluded^7^ that “... aqueous flow was more turbulent in models with higher TM/JCT stiffness.” Further, the report noted^7^ an “eddy or swirling of the aqueous outflow in the JCT and immediate vicinity of the µm-sized pores,” which “may explain the cause of a higher outflow resistance in eyes with stiffer outflow tissues.”

If turbulence were truly present in the outflow pathway, it would certainly influence outflow function and would have major consequences for outflow physiology, IOP mechanosensation and generation of flow resistance. However, recirculation, eddies and swirling flow do not require turbulence and, in fact, are commonly observed under conditions of laminar flow, particularly around obstacles. In other words, “eddy or swirling” features are not evidence for turbulence.^23^ Instead, the irregular motion characteristic of turbulence occurs at a critical value of a dimensionless quantity known as the Reynolds number^e^, which captures the relative importance of inertial to viscous forces for a particular flow field. Above a critical value of the Reynolds number, the flow transitions from an orderly laminar flow to a chaotic turbulent flow. This critical Reynolds number is approximately 2300 for flow in a tube.^23^

Two of the aforementioned studies^3^^,^^4^ reported an average Reynolds number of 0.28, whereas a third study^5^ reported a Reynolds number of 70. These values are well below the turbulent transition, and thus turbulence is not expected within the outflow pathway even based on these estimates. Moreover, these estimates of Reynolds number are orders-of-magnitude larger than prior literature estimates, which ranged from 7 × 10^−5^ in the TM^24^ to 2 × 10^−3^ in the pores of the inner wall of Schlemm’s canal.^25^^,^^26^ The elevated estimates of the Reynolds number are likely the consequence of supra-physiological outflow velocities in the pores. Using velocities that are consistent with outflow physiology leads to the conclusion that the Reynolds number throughout the outflow pathway is tiny (<<1), and there is no physical basis to postulate turbulence within the outflow pathway.

## Methodological Concerns

At a high level, the aforementioned studies^1^^–^^7^ follow the usual computational approach of solving the governing equations of fluid flow and tissue mechanics, allowing for coupled interactions between the fluid and solid components (often described as a “fluid-structure interaction” or FSI model). These solutions are then used to make quantitative predictions, such as the hydrodynamic shear stress acting on TM cells or the deformation experienced by TM beams. As with any computational approach, the validity of the computational predictions depends on the specification of several input parameters, including the detailed geometry, mechanical properties of the fluid and solid components, and boundary conditions. Erroneous input parameters, constraints, or conditions would call into question the validity of the computational results. Here, there are several areas of concern.

### Hydraulic Conductivity

One parameter the authors appear to specify is the “hydraulic conductivity,” which is inversely proportional to the resistance to aqueous humor flow through tissues. A hydraulic conductivity of “2.0 µL/min/mm Hg/cm^2^”^27^ was used to characterize the TM, whereas “2.5 mm Hg/µL/min/cm^2^”^28^ was used for the JCT, and “9000 × 10^−11^ cm^2^ seconds/g”^29^ for the inner wall of Schlemm’s canal.^1^^,^^3^^,^^6^^,^^7^ This approach raises several fundamental concerns.

• Specifying the hydraulic conductivity is inconsistent with also specifying the tissue ultrastructure, because the geometry and dimensions of the flow pathways, along with fluid viscosity, uniquely determine tissue flow resistance and thus the hydraulic conductivity. This is the reason why computational methods are normally used to *predict* the hydraulic conductivity based on the geometry of a given flow pathway.^28^^,^^30^^,^^31^ By assigning hydraulic conductivities to the various components of the outflow pathway, this approach essentially constrains the outcome from the computations. Indeed, figures from several of the aforementioned papers^1^^–^^3^^,^^6^^,^^7^ show a significant pressure gradient in the TM despite literature showing large open spaces in the TM,^32^^,^^33^ and that this region has negligible flow resistance.^29^^,^^34^
• Perkins et al.,^27^ cited as the source for establishing TM hydraulic conductivity, did not measure the hydraulic conductivity of the intact TM, but instead measured the hydraulic conductivity of a monolayer of “TM cells grown on filters.”^27^ One would expect a monolayer of cultured cells to have a far lower hydraulic conductivity than a porous three-dimensional meshwork lined by these same cells.
• Johnson^29^ cited as the source for the hydraulic conductivity of the inner wall, did not report that the hydraulic conductivity of the Schlemm’s canal inner wall is “9000 × 10^−11^ cm^2^ seconds/g.” Instead, he reported that the hydraulic conductivity “for the [entire] aqueous humor outflow pathway is between 4000 × 10^−11^ and 9000 × 10^−11^ cm^2^ seconds/g.” Assigning this range of hydraulic conductivity to the inner wall effectively attributes almost the entirety of outflow resistance to the inner wall.
• The hydraulic conductivity (or more accurately, the hydraulic “conductance”^35^) is defined as the ratio of the flow rate per unit area per unit pressure drop across a resistive tissue, and should have units of µL/min per mm Hg per cm^2^ or (expressed in cgs units) cm^2^ seconds/g. Thus, the units given for the hydraulic conductivity for the juxtacanalicular connective tissue (mm Hg/µL/minutes/cm^2^) are incorrect. Although this could be a typographical error, it is notable that it is repeated in four separate reports.^1^^,^^3^^,^^6^^,^^7^

### Schlemm’s Canal

The authors describe their hydrodynamic model of the lumen of Schlemm’s canal as “a compressible chamber” with properties set to that of air.^7^ This effectively introduces compressibility into what should otherwise be an incompressible flow. No rationale was given for such an extraordinary claim, nor any discussion of how compressibility would affect the computational results. It is unclear how many others of these papers used this compressible model of Schlemm’s canal, as no details were given on how Schlemm’s canal was modeled in the other manuscripts.^1^^–^^6^

### Aqueous Humor

The authors use a “Gruneisen equation of state with cubic shock velocity–particle velocity,”^4^^,^^5^ normally used to describe the pressures in a shock-compressed crystalline solid, to describe aqueous humor. This is extraordinary because aqueous humor is not a solid, nor a crystal, nor generally exposed to shock waves. Typically, aqueous humor is modeled as an incompressible fluid with properties like water. Although this may not necessarily lead to erroneous outputs, it is puzzling why such an equation of state would be needed for a model of physiological outflow.

### Application to Glaucoma

The authors use their model to claim that “the primary site of outflow resistance in healthy eyes was in the JCT and immediate vicinity of the SC [Schlemm's canal] inner wall, while the majority of the outflow resistance in the glaucoma eyes occurred in the TM.”^1^ This is a remarkable conclusion, which if true would have enormous consequences for our understanding of the pathophysiology of ocular hypertension in glaucoma. However, this model assumes that the size and density of pores in the inner wall endothelium were the same in both healthy and glaucomatous eyes, despite strong evidence that inner wall pore density is reduced in glaucoma.^13^^,^^36^ Furthermore, the investigators appear to assign the same values for the “hydraulic conductivity” of the TM, JCT, and inner wall between healthy and glaucomatous eyes, which is inconsistent with the known glaucomatous reduction in outflow facility.^37^ Stated differently, having specified that most of the resistance-generating outflow structures are the same between healthy and glaucomatous eyes, it is impossible to then use the model to investigate differences in resistance generation in glaucoma.

## Other Apparent Errors or Misrepresentations of the Literature

There are numerous other apparent errors or misrepresentations of the literature in the aforementioned papers.^1^^–^^7^ We mention a few of the more significant ones here.

• It was stated^1^ that “Ethier et al.^38^ and Stamer et al.^18^ reported the average theoretical shear stress of ∼0.1 to 1 Pa through the JCT to the SC inner wall (∼25 µm) using an idealized elliptical cross section.” This was used to support the computational shear stress results, stating^1^ “[t]he resultant shear stress in the FSI model (∼0.8–2.3 Pa) are closer to that of the Ethier and Stamer (∼0.1–1 Pa)^18^^,^^38^.” However, Ethier et al. and Stamer et al. did not model flow through the JCT but instead considered only flow and shear stress within the lumen of Schlemm’s canal. It makes no sense to use shear stress estimates for one anatomic region as a comparator for shear stresses in another region.
• It was stated^1^ that “McEwen showed that the trabecular space size of 12 µm in diameter in the healthy eyes^15^ that is in good agreement with our results (12.8 µm). Such a large diameter would drain the whole TM, so the trabecular space size of 5.7 [µm] in the glaucoma eyes would result in aqueous outflow resistance across the TM.” This is a misrepresentation of the McEwen report.^15^ McEwen provided a hydrodynamic calculation showing that a single pore that is 12 µm in diameter and 20 µm long could carry the entire aqueous humor flow at the normal physiologic pressure drop in the outflow pathway.^15^ There is no morphological work reported in McEwen’s paper. Instead, McEwen remarks that “It follows, therefore, that trabecular meshwork having interstices greater than 12 µm does not contribute to outflow resistance.”^15^ This does not necessarily imply the inverse, as suggested,^1^ that pores smaller than 12 µm are necessarily relevant to resistance generation.
• It was stated^5^ that “... it has been reported that there is no significant correlation between I-pore or B-pore density and post-mortem time.”^17^ This is not true. Swain et al.^17^ did not examine the relationship between pore density and postmortem time and only included two eyes in that study (making such a correlation meaningless).^17^ In contrast, Ethier et al.^14^ reported a significant decrease in I-pore density with postmortem time based on data from 34 eyes.^14^
• It was stated^4^ that “[a]nother study used finite element analysis to model the biomechanics of the outflow pathway and found that increasing the stiffness of the JCT and SC resulted in decreased outflow facility.^28^^,^^39^” Neither of these references examined tissue stiffness, and neither used finite element analysis.

## Summary

The series of computational studies described here^1^^–^^7^ represent an ambitious attempt to model the complex biomechanics of aqueous humor outflow. However, examination of their published body of work^1^^–^^7^ reveals what appear to be fundamental inconsistencies in computational outputs, including non-physiological values of outflow velocity and wall shear stress (see the Table), inappropriate specification of hydraulic conductivities, and questionable modeling choices. These concerns, combined with numerous misrepresentations of cited literature and inconsistencies between their own publications, undermine the validity of conclusions regarding outflow resistance, tissue biomechanics, and glaucoma pathophysiology. Given that these papers^1^^–^^7^ have appeared in journals that do not traditionally publish glaucoma research, yet contribute to the searchable literature, it is crucial to document our concerns before the ophthalmological community. We hope that doing so will stimulate critical discussion and inform future research, and we welcome a “letter to the editor” from the authors of these papers clarifying their thoughts on these issues.

## Footnotes

a To estimate the velocity (*v*) of aqueous humor flow in the TM of human eyes, consider a typical outflow rate (*Q*) of 2.5 µL/min (Ref. 8) and TM dimensions of 300 µm in the anterior-posterior direction (*h*) and a circumference (π *d*) of 36.4 mm, corresponding to an internal limbal diameter *d* of 11.6 mm (Ref. 9). Because a significant portion of the TM is occupied by impermeable materials such as cells and cell-lined beams, only a fraction of the total volume, known as the porosity ε, is available for fluid flow. The porosity is largest in the uveal meshwork and decreases as flow approaches the inner wall of Schlemm's canal. In the juxtacanalicular portion of the TM, for example, the porosity of perfusion-fixed non-glaucomatous human eyes is reported^10^ to be 37.4 ± 5.9%. Based on these factors, the flow velocity in the TM can be estimated as *v* = *Q*/(π *d* *h* ε). Using the values given above predicts a typical flow velocity in the JCT of roughly 10 µm/second.

b The total outflow rate can be estimated as *Q* = π *v* *d* *h* ε, as described in the footnote above.

c The average flow velocity through inner wall pores can be calculated as 4 *Q*/(*n* *A_iw_* π *D*^2^), where *n* = 835 pores/mm^2^ is the inner wall pore density, *A_iw_* = 11 mm^2^ is the inner wall area,^15^ *D* = 1.2 µm is the mean pore diameter, with a total conventional outflow rate *Q* of 2.5 µL/minute.

d Hydrodynamic shear stress can be estimated as *µv/D* where *µ* is the fluid viscosity, *v* the fluid velocity and *D* the length over which the velocity changes from its maximum value in the center of a flow passage to zero at a wall or boundary. Using a viscosity of aqueous humor of 7 × 10^−4^ Pa seconds,^21^ a typical velocity of 10 µm/seconds (see above), and an opening size in the JCT of approximately 2D ≈ 1 µm,^22^ yields an estimate of the wall shear stress of roughly 0.01 Pa. Alternatively, it is possible to use this relationship to estimate the length scale *D* that would be necessary to achieve a wall shear stress of 10 Pa with physiological flow velocities. D would need to be roughly 1 nm, which is a molecular length scale. These considerations indicate that shear stresses on the scale of 0.2 to 15 Pa (see the Table) are inconsistent with physiological flow rates and anatomic dimensions of the TM.

e The Reynolds number is computed as: $Re\;=\;\frac{\rho \nu D}{\mu }$ where ρ is the density of the fluid (1 g/cm^3^ for aqueous humor), μ is the viscosity of the fluid (0.007 g/cm/second for aqueous humor^21^), *D* is a characteristic size of the flow passage through which the fluid passes, and ν is the fluid velocity.
